# Bacteriophage PRD1 as a nanoscaffold for drug loading†

**DOI:** 10.1039/d1nr04153c

**Published:** 2021-12-01

**Authors:** Helen M. E. Duyvesteyn, Isaac Santos-Pérez, Francesca Peccati, Ane Martinez-Castillo, Thomas S. Walter, David Reguera, Felix M. Goñi, Gonzalo Jiménez-Osés, Hanna M. Oksanen, David I. Stuart, Nicola G. A. Abrescia

**Affiliations:** Division of Structural Biology, University of Oxford, The Henry Wellcome Building for Genomic Medicine Headington Oxford UK dave.stuart@strubi.ox.ac.uk; Diamond Light Source, Harwell Science and Innovation Campus Didcot UK; Structure and Cell Biology of Viruses Lab, Center for Cooperative Research in Biosciences (CIC bioGUNE), Basque Research and Technology Alliance (BRTA) Bizkaia Technology Park Derio Spain nabrescia@cicbiogune.es; Computational Chemistry Lab, CIC bioGUNE, Basque Research and Technology Alliance (BRTA) Bizkaia Technology Park Derio Spain; Departament de Física de la Matèria Condensada, Facultat de Física, Universitat de Barcelona Barcelona Spain; Departamento de Bioquímica, University of the Basque Country (UPV/EHU) and Instituto Biofisika (CSIC, UPV/EHU) Leioa Spain; IKERBASQUE, Basque Foundation for Science Bilbao Spain; Molecular and Integrative Biosciences Research Programme, Faculty of Biological and Environmental Sciences, Viikki Biocenter, University of Helsinki Helsinki Finland; Instruct-ERIC, Oxford House Parkway Court John Smith Drive Oxford UK; Centro de Investigación Biomédica en Red de Enfermedades Hepáticas y Digestivas (CIBERehd), Instituto de Salud Carlos III Madrid Spain

## Abstract

Viruses are very attractive biomaterials owing to their capability as nanocarriers of genetic material. Efforts have been made to functionalize self-assembling viral protein capsids on their exterior or interior to selectively take up different payloads. PRD1 is a double-stranded DNA bacteriophage comprising an icosahedral protein outer capsid and an inner lipidic vesicle. Here, we report the three-dimensional structure of PRD1 in complex with the antipsychotic drug chlorpromazine (CPZ) by cryo-electron microscopy. We show that the jellyrolls of the viral major capsid protein P3, protruding outwards from the capsid shell, serve as scaffolds for loading heterocyclic CPZ molecules. Additional X-ray studies and molecular dynamics simulations show the binding modes and organization of CPZ molecules when complexed with P3 only and onto the virion surface. Collectively, we provide a proof of concept for the possible use of the lattice-like organisation and the quasi-symmetric morphology of virus capsomers for loading heterocyclic drugs with defined properties.

## Abstract 2

Viruses are very attractive biomaterials owing to their capability as nanocarriers of genetic material.

## Graphical abstract

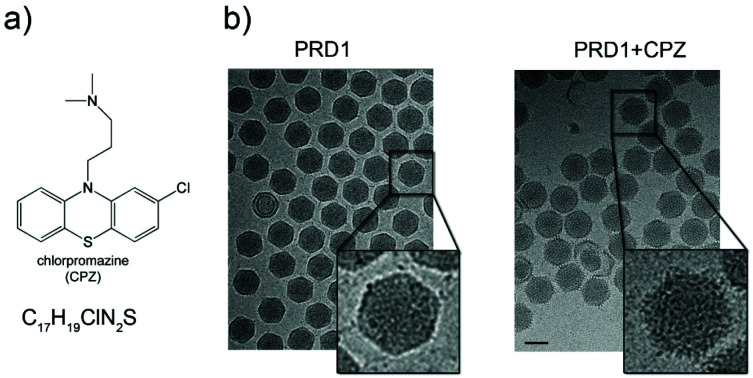

## Introduction

Viruses have been targeted for their potential as nanocarriers of drugs or repairing genes for decades.^1–3^ A viral capsid can form a protective layer over a given drug formulation and may also be engineered for site-specific release.^4–6^ Plant viruses and bacteriophages have been used as workbenches for nanotechnology and therapeutic developments.^7–10^ In addition, minimal biosecurity concerns and extensive knowledge facilitate the selection and design of phage candidates.

Icosahedral membrane-containing bacteriophage PRD1, a model system for the family *Tectiviridae* to which it belongs, and more generally for complex double-stranded DNA viruses, is representative of the PRD1-adenovirus structure-based lineage (the kingdom *Bamfordvirae*). Viruses belonging to this kingdom possess major capsid proteins (MCPs) adopting a double jellyroll fold.^11,12^ The 66 MDa PRD1 virion comprises the MCP P3 protein (43 kDa) arranged into 240 trimeric units (capsomers with pseudo-hexameric morphology) on the icosahedral lattice. Lattice vertices are occupied by P31 pentamers that form the base for the flexible spike complex formed of the trimeric P5 protein and the receptor binding protein P2.^13^ Underneath the capsid there is the membrane vesicle whose remodelling leads to a tubular structure protruding from the unique vertex used to infect the host cell.^14–16^

During investigations into the role of viral membranes in assisting viral DNA ejection,^17^ the phenothiazine compound chlorpromazine (CPZ)‡CPZ-PRD1 cryo-EM map has been deposited in the Electron Microscopy and Protein Data Bank, code: EMD-13109. The crystal structure of the MCP P3-CPZ complex has been deposited in the PDB with code: 7OOK. was selected as a probe of viral membrane stability due to its known ability to destabilise the liposomal structure.^18–20^ However, we found that attempts to infuse CPZ at different concentrations into PRD1 particles resulted in the decoration of the outer capsid. CPZ is a moderately basic [p*K*_a_ 8.6] amphipathic drug (drugbank ID DB00477) with an ability to form micelles depending on environmental conditions.^21,22^ It binds dopamine D1–D5 and histamine receptors and has been historically approved to treat mental disorders including schizophrenia.^23^ Access to the central nervous system is achieved through binding to erythrocytes. However, CPZ's use has been superseded by alternative candidates owing to its hepatotoxicity in some patients.^24^ Nevertheless, phenothiazine compounds are model lead structures in medicinal chemistry serving as molecular templates for developing pharmacotherapeutics.^25^

In this study, we demonstrate PRD1's potential as a nanocarrier for CPZ. Using cryo-electron microscopy (cryo-EM), X-ray crystallography, biochemical and molecular dynamics (MD) techniques, we show that CPZ molecules, and related phenothiazine derivatives, accumulate at specific locations on the phage capsid because of: (i) CPZ's capacity for micellization; (ii) the highly polar *N*,*N*-dimethylpropylamine moiety in CPZ interacting with the negatively charged residues at the apex of one of the two P3 jellyrolls; (iii) the turreted morphology resulting in the P3 capsomers arranging into a closely packed hexagonal lattice with local p3 symmetry on each icosahedral facet. However, these interactions do not hinder infectivity or virion stability. When soaked in excess into P3 crystals, CPZ aromatic rings stack between the indole ring of a tryptophan side chain and the guanidinium group of an arginine of an adjacent P3, located at mid-height of the molecule.

Our findings represent a proof of concept for the use of PRD1's lattice and capsid morphology for loading heterocyclic drugs without the need for genetic or biochemical modifications of virions.

## Results and discussion

### Overall morphology of PRD1 in the presence of chlorpromazine

**Fig. 1:**
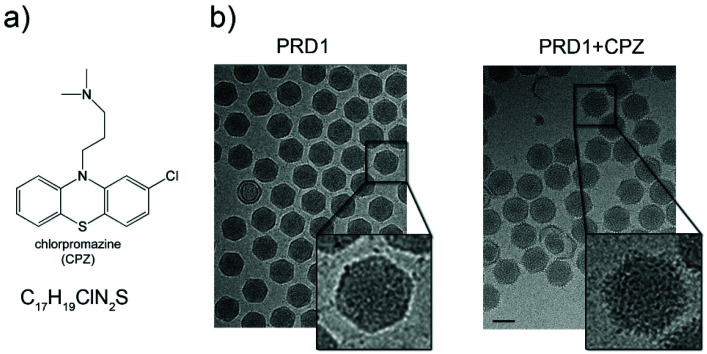
Chlorpromazine and its effects on PRD1 morphology. (a) Chlorpromazine (CPZ) structure and molecular formula. (b) Left, wild-type (wt) PRD1 sample as visualized by 2D cryo-EM; right, PRD1 morphology when mixed with CPZ (see also Fig. S1a†). 2D cryo-EM images are to scale; scale bar, 50 nm.

Purified PRD1 was mixed with increasing concentrations of CPZ (2–60 mM) before application to cryo-EM grids for imaging (Fig. 1 and Fig. S1a†; see Experimental†). The critical micelle concentration (CMC) of CPZ is dependent on buffer pH and ionic strength and decreases with increasing pH from ∼2 mM at pH 5.6 to ∼0.2 mM at pH 7.3.^21^ Cryo-electron micrographs showed a stark difference between CPZ-PRD1 particles and control particles without CPZ, with the PRD1 capsids mixed with increasing CPZ concentration having a ‘spiky’ appearance in contrast to the smooth surface of control particles (Fig. 1b and Fig. S1a†). This spikey appearance persisted following dialysis in a CPZ-free buffer for two hours, implying that this decoration was irreversibly bound within that timeframe and buffer conditions (Fig. S1a†).

**Fig. 2:**
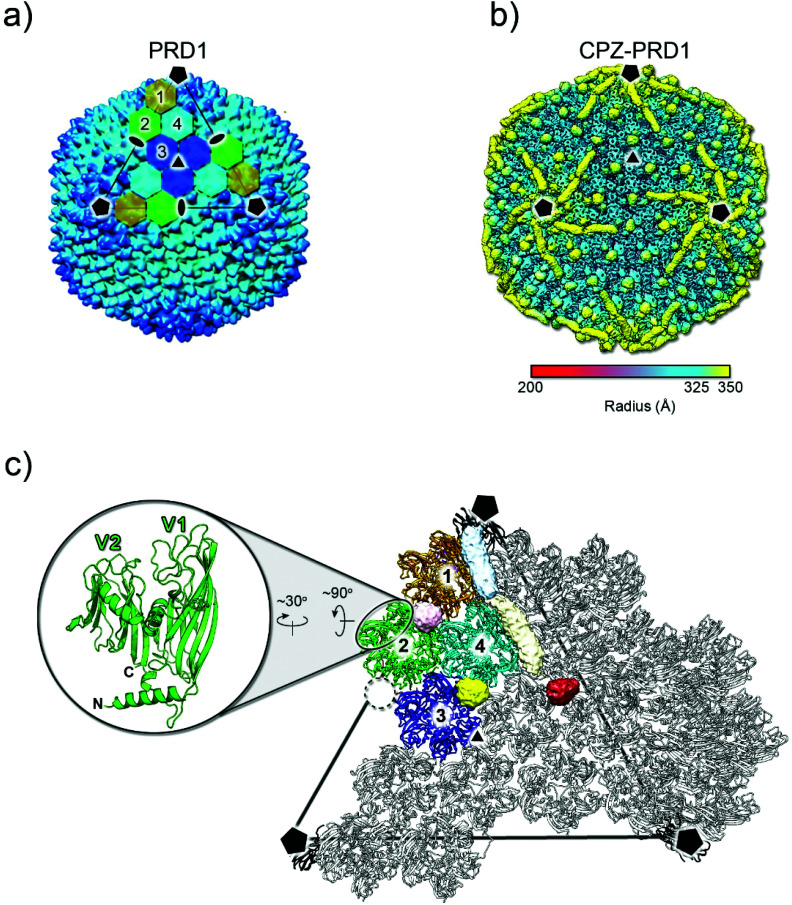
3D structure of CPZ-PRD1 complex. (a) Overall morphology of PRD1 phage derived from the atomic model (PDB ID 1w8x). The black lines mark one of the icosahedral facets of the virion and the black pentagons, triangle and ovals identify the 5-, 3- and 2-fold icosahedral symmetry axes, respectively. The icosahedral asymmetric unit is composed of four pseudo-hexameric capsomers coloured in ochre, light-green, cyan, and blue. Capsomers are formed by the trimerization of MCP P3 (see below). (b) Density of the CPZ-PRD1 complex determined by cryo-EM, Gaussian filtered with a width of 1.39 Å, displayed at 0.018 threshold (2.7 times the map rms) in Chimera^39^ and colour-coded by radius as from legend [symbols as in (a)]; for a lower threshold rendering of the map, please see Fig. S1b.† (c) Location of the independent extra densities due to the presence of CPZ; in yellow, light-pink and red those with sphere-like shapes while in light-blue and beige the cigar-like ones. At 0.018 threshold, the sphere-like densities display a diameter of ∼14 Å while the cigar-like densities display a minor and major axis of ∼14 and ∼40 Å, respectively. The dashed black circle marks the position where the spherical red extra-density is located relative to the capsomers 1–4 composing the icosahedral asymmetric unit [coloured as in (a) while adjacent ones in grey]. The inset shows the double jellyroll fold adopted by the MCP P3 depicted in green cartoon with V1 (residues 1–245) and V2 (residues 246–383) labelling the individual jellyrolls.

The 3D reconstruction of the cryo-electron micrographs of CPZ-PRD1 particles led to a 3.9 Å resolution density map‡ as estimated by the Fourier shell correlation (FSC) at the 0.143 threshold criterion (Fig. S2 and Table S1†). Relative to the previously published PRD1 apo structure (PDB ID 1w8x), the CPZ-PRD1 cryo-EM map showed the familiar pseudo-hexameric capsomers building the capsid, but with additional features decorating the exterior of the capsid layer (Fig. 2a and b). The extra-densities display distinct shapes and locations relative to the capsomers; sphere-like densities sit across the top of the β-loops of three contiguous V1 jellyrolls of MCP P3 whilst cigar-like densities snake their way from the five-fold, nearby a peripentonal V1 jellyroll, towards the two-fold wedging across two adjacent V1 jellyrolls belonging to the capsomers of neighbouring facets (Fig. 2b, c and Fig. S1b†). Fitting of the published PRD1 crystallographic model into the cryo-EM density indicated no significant structural changes in the MCP fold and no appreciable structural changes in the interior of the virion (Fig. 2c). CPZ is the only additional component and the features are not sufficiently well resolved to permit fitting of the individual CPZ molecules, although their precise location onto the icosahedral capsid denotes specific interactions with the jellyroll towers of MCP P3 (Fig. 2c).

### CPZ does not significantly affect virus infectivity or compromise virus stability

**Fig. 3:**
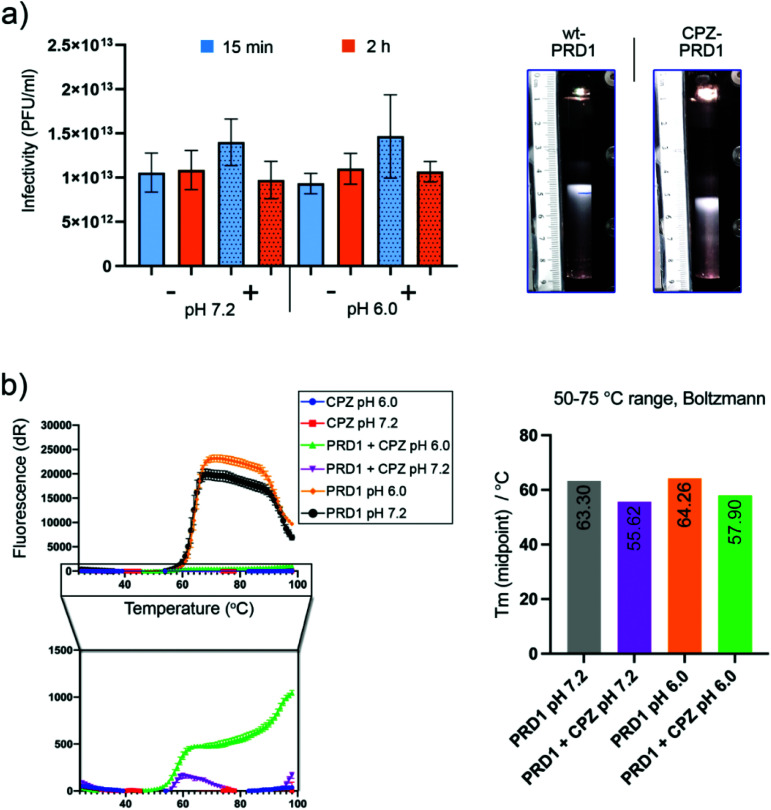
Infectivity and thermostability assays of CPZ-PRD1. (a) Left, virus infectivity in the presence of CPZ. Purified particles were treated with 60 mM CPZ in 20 mM potassium phosphate pH 7.2 or pH 6.0, 1 mM MgCl_2_ for 15 min or 2 h at room temperature after which the infectivities were determined. Right, light scattering particle zones after CPZ-treatment (30 mM CPZ at pH 6.0, 15 min) and sedimentation in CPZ-free sucrose gradient (ultracentrifugation). Untreated control (wt; typical sedimentation of wt PRD1) and CPZ-PRD1. (b) Left, first derivative curve from thermofluor dataset with PRD1 and CPZ at pH values 6 and 7.2. The signal was quenched in the presence of CPZ. Right, midpoints derived from Boltzmann-fitted curves for PRD1 incubated with CPZ in the temperature range of 50–75 °C. Midpoints in °C are displayed on each bar.

To assess CPZ's impact on PRD1 infectivity and stability, infectivity and sedimentation assays were carried out. No significant loss of infectivity was observed relative to a CPZ-free control after two hours at pH 7.2 or pH 6.0 (Fig. 3a, left). CPZ-treated PRD1 was found to sediment faster than CPZ-free controls forming a light-scattering band (Fig. 3a, right) or accumulated at the bottom of the tube in a run in which the CPZ-free particle sedimented as wild-type PRD1, further evidencing that CPZ irreversibly decorates the virions likely by favouring aggregation.

After rate zonal sedimentation, the specific infectivity of CPZ-PRD1 (1.1 × 10^12^ PFU/A280) was almost at the same level as that of CPZ-free PRD1 (1.3 × 10^12^ PFU/A280) in the bands (Fig. 3a, right). Analysis of the samples from rate zonal centrifugation showed that the protein composition of CPZ-PRD1 was the same as that of the non-treated one (Fig. S3†). The marginal change in infectivity (Fig. 3a, left) may be a consequence of CPZ quenching and particle aggregation, the latter consistent with observations during cryo-grid screening, where particles were found to form particle swarms. These observations support the hypothesis that the PRD1 capsid works as an inert scaffold for drug loading.

To assess whether increasing the ionic strength would perturbate the interaction between PRD1 and CPZ molecules, PRD1-CPZ samples with 150 or 300 mM NaCl were visualised by cryo-EM and tested by rate zonal sedimentation. Both experiments suggest that the presence of NaCl at these concentrations does not revert the binding, but rather influences the aggregative properties of the sample (Fig. S4†).

Furthermore, to probe CPZ's impact on capsid thermostability, a thermofluor assay was conducted using a genome-sensitive dye in triplicate and at pH 6.0 and pH 7.2 (Fig. 3b). CPZ was found to have a slight quenching effect (this was confirmed by nanodrop, fluorescence emission peak ∼478 nm; maximum excitation peak ∼376 nm. Nevertheless, a reduction of about 8 °C in the melting temperature was observed upon incubation with CPZ. This could be interpreted as CPZ penetrating deeper into the capsid following increased capsid porosity upon heating causing destabilisation of the inner membrane (Fig. 3b, right). But the substantially reduced signal due to CPZ quenching necessitates cautious interpretation.

### Interaction of CPZ with the crystal structure of the major capsid protein P3

**Fig. 4:**
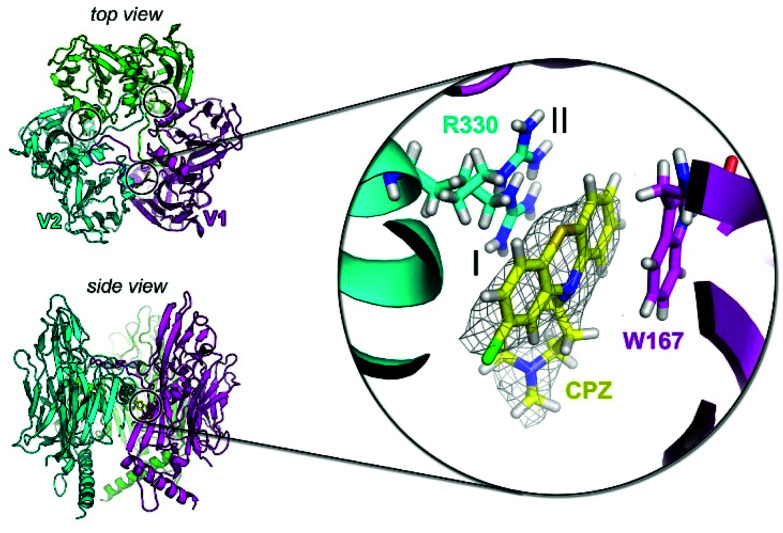
P3 crystal structure in complex with CPZ. Top left, view from the top of the crystallographic trimer depicted in cartoon with each P3 monomer coloured in green, cyan and magenta. The three black circles mark the location of the three bound CPZ molecules and V1 and V2 define the two contiguous jellyrolls of distinct P3 monomers. Bottom left, view from the side of the P3 trimer. Inset, structure of the CPZ binding pocket with the 2Fo–Fc electron density map (grey mesh) of one of the CPZ molecules (in yellow stick) contoured at 0.8*σ* and nearby the interacting residues W167 and R330; the latter in double conformation (I, occupancy = 0.5; II, occupancy = 0.5).

To investigate the potential of specific binding of CPZ to individual P3 molecules, P3 was crystallised using published conditions^26^ and soaked with 1 mM, 15 mM and 25 mM CPZ. Crystals diffracted to 2.3 Å resolution (Table S2†). Within each crystallographic asymmetric unit, P3 forms a trimer (Fig. 4) that replicates the pseudo-hexameric capsomers used by the virion as the fundamental building block for assembly^10,23^ (Fig. 2c). The initial, ligand-free refinement showed clear density for CPZ at the interface of contiguous P3 molecules between the BIDG and CHEF walls of jellyrolls V1 and V2, belonging respectively to different monomers, at concentrations of 15 mM and above (Fig. 4). For the apo structure, the density was consistent with a 2-methyl-2,4-pentanediol (MPD) chemical additive (previously modelled in the deposited crystal structure with PDB ID 1hx6^26^), but this feature became more elongated for the CPZ soaks at 15 mM and above. The CPZ molecule fitted well into this extended MPD site, with the CPZ located in a hydrophobic pocket and sandwiched between residue W167 of one P3 molecule forming π–π stacking interactions and the guanidinium group of residue R330 of another monomer, with the latter residue displaying a double conformation (Fig. 4 zoom-in). CPZ molecules were found in the three equivalent positions on the trimeric P3 and no secondary binding locations were detected.

As a model drug for thiazine scaffolds, CPZ has been previously co-crystallised with human proteins such as S100B and α(1)-acid glycoprotein (hAGP), and with the Erwinia ligand-gated ion channel, a prokaryotic GABA-gated receptor.^27–29^ In the latter case, unexpectedly, CPZ was not found at the pore site but near a β-loop stabilised mainly by hydrophobic interactions and responsible for receptor inhibition.^29^

### CPZ distribution on the PRD1 capsid

Signal contributed by any viral proteins or ligands disobeying imposed 60-fold symmetry is greatly diminished during the averaging process of 3D icosahedral reconstruction. Thus, the extra-densities decorating the virion particles are assumed to reflect an average of locations at which the CPZ molecules fulfil the icosahedral symmetry (Fig. 2b). However, the spherical and elongated densities within the icosahedral asymmetric unit might still be the result of the different loading modes of the CPZ molecules at those same equivalent positions across the entire virions (Fig. 2c and Fig. S5a†). Indeed, early crystallographic studies of CPZ alone showed different packing arrangements of the compound depending on the presence of a counter-anion or on a protonation state^30–32^ (Fig. S5b and Table S3†).

**Fig. 5:**
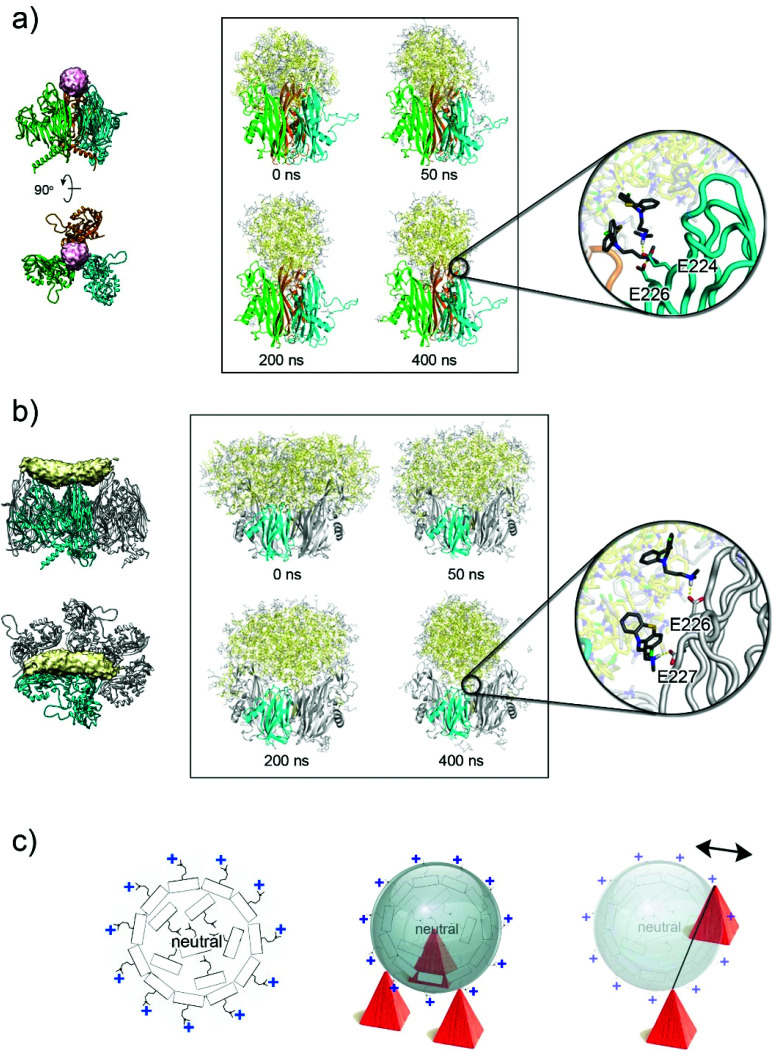
CPZ accumulation on the virion's capsid. (a) Left, location of the spherical extra-density (in pink) within the three apices of the V1 jellyrolls (as cartoon coloured in cyan, green and orange) of three different P3 molecules. Right, side-by-side comparison of the CPZ molecules (in yellow, neutral CPZ; in grey, charged CPZ) modelled by MD simulations at different times, the latter showing the reorganization of the molecules; the V2 jellyrolls and the water molecules are not depicted for clarity. Inset shows the details of the interaction of the first protonated shell of CPZ molecules with the negative charged residues present on the V1 jellyroll depicted as a cartoon-tube (see Table S4†). (b) As (a) but for the elongated density (see Table S5†); P3 residues 36–239 and 257–381 and the water molecules are not depicted for clarity. (c) Schematic diagram (left) of the supramolecular complexes formed by CPZ molecules as obtained from MD simulations with the external layer positively charged due to the highly polar *N*,*N*-dimethylpropylamine moiety pointing outwards and the interior filled with neutral CPZ molecules irregularly arranged; middle, schematic representation of a CPZ micelle interacting with the negatively charged three-fold pseudo-symmetric P3 V1 jellyrolls represented as red pyramids. Right, as middle but depicting graphically the context of the V1 and V2 jellyrolls of contiguous P3 (only V1 jellyroll shown as red pyramids) with on top a micelle misaligned relative to the hypothetical line joining the two V1 jellyrolls (the double-headed arrow indicates the putative movements of the micelles).

To evaluate the possible origin of these extra-densities and the arrangement of the CPZ molecules within them, we used MD simulations. For each simulation, clusters of CPZ molecules, with previously quantum-mechanically optimized geometries, were placed in the context of different regions of P3. In the case of spherical density, this region comprises three V1 jellyrolls of P3 monomers related by a pseudo 3-fold symmetry axis; for example, those across capsomers 1–2–4 are shown in Fig. 2a, c and Fig. 5a. For the elongated density, the V1 and V2 jellyrolls of contiguous P3 along the edge of the icosahedral asymmetric unit were selected (Fig. 5b). Electrostatic potential analysis of the P3 capsomers has shown that there is a clear segregation in charge distribution between the interior and exterior sides of the capsomers with the V1 jellyroll displaying a marked negative charge on the exteriors^33^ (Fig. S5c and d†). These negative patches would engage the positively charged *N*,*N*-dimethylpropanammonium tail of CPZ through noncovalent interactions. In addition, CPZ has a p*K*_a_ of 8.6, meaning that its protonated (charged) and deprotonated (neutral) forms will coexist at the experimental condition of pH 7.2.^21^ Indeed, MD simulations of clusters of 100% charged CPZ molecules in water solution indicated the distribution of CPZ molecules quenching the possible binding sites on the capsid (Fig. S5e†). For the cavity corresponding to the spherical density, over a simulation of 400 ns and using a 50 : 50 mixture of charged and neutral molecules, many CPZ molecules remain trapped between the P3 charged residues, *e.g.* E224, E226, and E227 along the β-strands of the V1 jellyrolls, whose proximity and relative orientation favour CPZ clustering (Fig. 5a and Table S5†). The central CPZ cluster coordinates were fitted to a spherical surface along evenly sampled MD snapshots using an in-house code and the evolution of the molecules in/out from the surface along the simulation was analysed (Fig. S6 and Movie S1†). Water molecules are progressively excluded from the CPZ cluster with significant shrinking of the aggregate. Likewise, charged CPZ molecules migrate to the cluster surface leaving a densely packed core of mostly neutral CPZ (diameter ∼ 50 Å).

A similar CPZ behaviour and organization was observed when clusters of CPZ molecules were placed in the context of the V1 and V2 jellyrolls of contiguous P3. After 400 ns simulation, the CPZ aggregate adopted a more spherical shape (diameter ∼60 Å) rather than the averaged observed elongated cigar-like density (Fig. 5b, and Fig. S7, Table S5 and Movie S2†). In this case, however, the morphology of the protein scaffold lacks the tighter constraints generated by the pseudo three-fold symmetric V1 jellyrolls subjacent the sphere-like extra-densities.

In both scenarios, the MD results indicate the propensity of the CPZ molecules, when framed within different proteinaceous scaffolds, to converge into spherical supramolecular complexes but with a different degree of penetration within each of the proteinaceous moulds as dictated by the more or less proximity of negatively charged residues (Fig. 5a and b, at 400 ns and Fig. 5c).

So, how can the cigar-like shaped density be accounted for?

A possible explanation is that the cigar-like density is the averaged density resulting from the sphere-like CPZ complexes misaligned from a hypothetical axis joining the two opposing V1 jellyrolls (Fig. 5c, right). To address this hypothesis, we analysed the aggregate mobility along MD simulations computing the squared atomic positional fluctuations of CPZ molecules and weighting them by (8/3)π^2^ (*b*-factors). Results for the system representing the cigar-like density show an elongated shape for the distribution of the least mobile fraction of CPZ molecules, with an accumulation of low-mobility CPZ in the proximity of the V1 jellyrolls (Fig. S8a†). This asymmetric mobility distribution is likely due to the favourable interaction with the charged residues of the V1 jellyrolls, which stabilizes neighbouring CPZs and confers the elongated shape to the extra density. The observed cigar-like densities contact individual V1 jellyrolls distanced along the edge of a facet with only two V1, related by a pseudo two-fold symmetry, clamping both sides of the cigar-densities (near the interface between the pale-cyan and pale-yellow densities; Fig. 2c)—these discrete contacts may confer more dynamism to the CPZ micelles.

However, alternative and/or complementary scenarios can occur where heterogeneously sized pre-formed CPZ supramolecular complexes could be sequestered by the highly polar V1 towers. The MD simulations of CPZ alone with different mixtures of charged and neutral molecules show that with the increase of charged molecules the size of the micelles formed decreases (Fig. S8b†). In this scenario, differently sized CPZ complexes would dock according to the local molecular architecture and electrostatic properties of the virion capsomers (Fig. 5c).

Cryo-EM images of CPZ alone at about 30 mM concentration showed a distribution of apparent black dots with a roughly estimated diameter of 20–40 Å and within the range of the diameter and shorter axis of the aforementioned spherical and elongated densities, respectively (Fig. 2b and Fig. S1,† inset).

The density of CPZ molecules in both the extra regions was estimated from the MD simulations, obtaining values of 1.25 ± 0.10 g cm^−3^ and 1.17 ± 0.05 g cm^−3^ for the sphere-like and cigar-like extra-densities, respectively. These values are only slightly less than the density of 1.28 g cm^−3^ that is calculated for the crystallographic structure of pure CPZ in the orthorhombic *Pbca* space group, which are likely due to the amorphous (*i.e.* non-crystalline) nature of the micelle-like aggregates.^30^ In terms of the number of CPZ molecules decorating PRD1, at the threshold used to contour the density as in Fig. 2b, we conservatively estimate at least 1500 molecules per virion assuming ∼3 and ∼8 CPZ molecules in the spheric and cigar-like densities, respectively (see Experimental†).

In light of these results, we also tested by 2D cryo-EM imaging two additional compounds, namely promazine hydrochloride (PMZ; drugbank DB 00420) and *N*,*N*-dimethyl-3-(10*H*-phenoxazin-10-yl)-1-propanamine (DPP hereafter; ChemSpider ID 173635) (Fig. S9†). The former lacks the chlorine atom, while the latter has an oxygen atom replacing the sulphur atom conferring increased planarity to the heterocycle. When PMZ [CMC = 35.05 mM^34^] was mixed with PRD1, only the mixture with 30 mM PMZ showed the distinguishable presence of aggregates also decorating the PRD1 capsid (Fig. S9a†). A similar effect was observed when DPP was used although its CMC is not known. In both the cases, the effect of high concentration (*e.g.* 30 mM) on the virion is qualitatively more drastic compared to that of CPZ, as broken particles are also apparent (Fig. S9b†).

Thus, the origin of the decorated capsid is the result of the physicochemical properties of CPZ, the negatively charged residues close-by the apex of the V1 jellyroll and the relative spatial arrangement of the capsomers on the assembled virion. The design of the PRD1 viral capsid serves as a scaffold for loading the CPZ molecules likely pre-assembled as micelles and/or with the capability to self-assemble as also shown by the other phenothiazine compounds (Fig. S9†). Specifically, it is the turreted morphology resulting from the double jellyroll fold of the MCP P3 and from the relative orientation of the contiguous apices of the V1 jellyroll that confer not just a one point/residue interaction but a series of nearby negatively charged anchoring points acting as a ‘catch cone’ that ‘trap’ the CPZ molecules/micelles (Fig. 2C). While CPZ molecules/micelles could, in principle, interact with negatively charged patches on the surface of other viruses with different capsid protein folds, it remains to be seen whether this interaction would be stable enough to organize the CPZ in an orderly fashion around the virion. Thus, the PRD1 double jellyroll is not special ‘per se’, indeed the same fold is shared across many viruses—of course with different charge distributions on their coat protein, but the lattice with the towers of the jellyroll will be preserved across all the other viruses (some with a higher tower than others), offering a similar ‘morphological landscape’ for the potential anchoring of CPZ molecules. The PRD1 Sus1 procapsid would be of particular interest for such a storage scaffold since it lacks the viral genome but retains the electrostatic properties of the wild-type capsid and has similar mechanical stability.^35^ In the past, plant-infecting viruses such as red clover necrotic mosaic virus and cowpea mosaic virus have been targeted for a non-covalent infusion technique which consists of filling the virion's interior with drugs.^36,37^

## Conclusions

PRD1 is a representative of the large and expanding PRD1-adenovirus viral lineage, recently recognised as defining a viral kingdom; the members of this lineage share the double jellyroll fold in their major capsid proteins and replicate the capsid design.^38^

Here, we explain the serendipitous observation that enterobacteriophage PRD1 capsids become decorated in the presence of CPZ at concentrations above its CMC. We show that this decoration is the result of sequestration of CPZ molecules by the PRD1 particle acting as the attractor and/or inducer of small micelles and this ‘loader’ capability is extensible to other members of the phenothiazine class of agents. Although further studies are required to assess the applicability of this method for cargo-loading and delivery, the present study provides a proof of concept for exploring viruses harmless to humans serving as drug carriers by solely exploiting their capsid electrostatic properties and scaffolding capability without the need for genetic engineering or chemical modification.

## Author contributions

NGAA conceived the study with input from DR. ISP and AM-C performed sample preparation and 2D cryo-imaging. HMED and TSW conducted fluorescence and crystallographic experiments with NGAA finalizing the crystal refinement. HMED performed sample preparation, cryo-EM data collection and 3D reconstruction. FP and GJ-O undertook MD simulations. HMO provided the purified P3 protein for crystallization and bacteriophage PRD1 for cryo-EM and performed infectivity studies. FMG and DIS provided conceptualisation and infrastructure. NGAA supervised the multidisciplinary study. All authors analysed the data and contributed to the finalization of the manuscript.

## Conflicts of interest

There are no conflicts to declare.

## Supplementary Material

NR-013-D1NR04153C-s001

NR-013-D1NR04153C-s002

NR-013-D1NR04153C-s003
