# Supplementary material for: Bacteriophage PRD1 as a nanoscaffold for drug loading
Source: Nanoscale. 2021 Dec 1;13(47):19875–83. doi: 10.1039/d1nr04153c (PMC8667075; doi:10.1039/d1nr04153c)
Supplement: NR-013-D1NR04153C-s001 [file NR-013-D1NR04153C-s001.pdf]

ELECTRONIC SUPPLEMENTARY INFORMATION (ESI)

Bacteriophage PRD1 as a nanoscaffold for drug loading

Helen M E Duyvesteyn [a,b], Isaac Santos-Pérez [c], Francesca Peccati [d], Ane Martinez-Castillo [c], Thomas S. Walter [a], David Reguera [e], Felix M Goñi [f], Gonzalo Jiménez-Osés [d,g], Hanna M Oksanen [h], David I Stuart [*a,b], Nicola GA Abrescia [*c,g,i]

Experimental

Virus and major capsid protein P3 production and purification

Lipid-containing bacteriophage PRD1 was produced and purified as previously described[40]. Briefly, wild-type (wt) PRD1 was propagated on *Salmonella enterica* serovar Typhimurium strain DS88 and cells were infected using a multiplicity of infection of 8–10. Virus particles were purified by polyethylene glycol–NaCl precipitation and rate zonal and equilibrium ultracentrifugation in sucrose (Sorvall rotor AH629). Purified virus particles were shipped to Bilbao (Spain) or Oxford (UK) for cryo-EM structural studies. The P3 MCP was purified directly from the virus accordingly to the established protocol[26,41].

Sample preparation for cryo-EM

Purified PRD1 sample in 20 mM potassium phosphate buffer pH 7.2 with 1 mM $MgCl_2$ was mixed with Chlorpromazine-HCl (CPZ-HCl; Sigma-Aldrich Ref. C8138; molar mass: 355,33 g/mol) previously dissolved in the same buffer to give a ~42.6 mg/ml (120 mM; ~72.3 x 10^{21} molecules per ml) concentrated solution. The explored protein concentrations of the PRD1 sample ranged between 1.0 and 0.2 mg/ml and the concentration of CPZ from 0.7 mg/ml (2 mM) to ~21.3 mg/ml (60 mM). Samples of the CPZ-PRD1 mixture were incubated for 15 min at room temperature before application to R2/2 and/or R1.2/1.3 Quantifoil copper grids and vitrification in liquid ethane using a Vitrobot (FEI Mark III) and/or an EM GP2 (Leica Microsystems) automatic plungers. All CPZ concentrations used were similar or above the estimated CMC[21,22]. By cryo-imaging, 'spikes' were increasingly visible at higher CPZ concentrations when inspected by cryo-EM (Fig. 1b and Fig. S1a†).

A similar procedure was followed for the dialysed sample with the difference that the CPZ-PRD1 mix (250 µl of ~0.3 mg/ml of PRD1 *plus* 250 µl of ~60 mM CPZ) was first loaded into a Float-A-Lyzer G2 dialysis device (1 ml volume, MWCO 1000 kDa; Spectra/Por) and left for 2 hours at 4 °C equilibrating in agitation against 2 L solution of 20 mM potassium phosphate pH 7.2 and 1 mM $MgCl_2$ before further use (Fig. S1b†).

Samples of PRD1 with promazine hydrochloride (PMZ-HCl; Sigma-Aldrich Ref. P6656; molar mass: 320.88 g/mol) and *N,N*-dimethyl-3-(10H-phenoxazin-10-yl)-1-propanamine (DPP; Sigma-Aldrich; Ref. PH008159; molar mass: 268,36 g/mol) at concentrations of 30, 10 and 2 mM, respectively, were similarly prepared as the non-dialysed samples and vitrified for subsequent 2D cryo-imaging (Fig. S8†).

Cryo-EM data collection

2D cryo-images of CPZ-PRD1, PMZ-PRD1 and DPP-PRD1 samples were acquired at the CIC bioGUNE (Spain) using a JEOL JEM-2200FS/CR 200 kV microscope equipped with an omega in-column energy filter and a K2 Summit (GATAN) camera in linear mode at 30,000 X nominal magnification, corresponding to 1.3 Å/pix at the specimen and using underfocus values between 2.5-3.0 µm.

[a] Address here. Division of Structural Biology, University of Oxford, The Henry Wellcome Building for Genomic Medicine, Headington, Oxford, UK. E-mail: dave.stuart@strubi.ox.ac.uk
[b] Diamond Light Source, Harwell Science and Innovation Campus, Didcot, UK.
[c] Structure and Cell Biology of Viruses Lab, Center for Cooperative Research in Biosciences (CIC bioGUNE), Basque Research and Technology Alliance (BRTA), Bizkaia Technology Park, Derio, Spain. E-mail: nabrescia@cicbiogune.es
[d] Computational Chemistry Lab, CIC bioGUNE, Basque Research and Technology Alliance (BRTA), Bizkaia Technology Park, Derio, Spain.
[e] Departament de Física de la Matèria Condensada, Facultat de Física, Universitat de Barcelona, Barcelona, Spain.
[f] Departamento de Bioquímica, University of the Basque Country (UPV/EHU) and Instituto Biofisika (CSIC, UPV/EHU), Leioa, Spain.
[g] IKERBASQUE, Basque Foundation for Science, Bilbao, Spain.
[h] Molecular and Integrative Biosciences Research Programme, Faculty of Biological and Environmental Sciences, Viikki Biocenter, University of Helsinki, Helsinki, Finland.
[i] Instruct-ERIC, Oxford House, Parkway Court, John Smith Drive, Oxford, UK.
[j] Centro de Investigación Biomédica en Red de Enfermedades Hepáticas y Digestivas (CIBERehd), Instituto de Salud Carlos III, Madrid, Spain.

Please do not adjust margins

Due to significant aggregation of PRD1 particles in the presence of CPZ, sample and grid optimization were essential steps prior to data collection for high-resolution 3D reconstruction (sample concentration: ~1 mg/ml of PRD1 and ~60 mM of CPZ). Movies were recorded at the OPIC facility at the Division of Structural Biology at Oxford University-WHG (UK) on a Titan Krios microscope (ThermoFisher FEI) with a Falcon III camera in linear mode and a nominal magnification of 59,000 X, calibrated to 1.39 Å/pix (Table S1†).

Image processing, 3D reconstruction and model refinement

Movie pre-processing was performed using MotionCorr2.1[42], ctf-corrected with CtfFind4.2[43]. All subsequent processing was performed using RELION 2.0 and RELION 3.0[44]. Owing to the high density and substantial overlap of virions conventional software failed to correctly distinguish an adequate number of particles; particles were instead manually selected using the RELION 2.0 interface. Extracted virions were then subjected to 2D and then 3D classification with icosahedral symmetry imposed and using a reference volume derived from the published PRD1 structure (PDB ID 1w8x)[13]. After a 3D auto-refine job, three rounds of CTF refinement were then performed using RELION 3.0. This resulted in a final post-processed electron density map exhibiting a 3.9 Å resolution as estimated by the gold-standard FSC (0.143 threshold) within a threshold mask (10 Å low-pass filter, 0.02 initial binarisation threshold, 5 pixels extension of the binary map plus 5 soft-edge pixels) (Fig. S2†).

The P3 MCPs making up an icosahedral asymmetric unit (PDB ID 1w8x) were fitted into the corresponding density using CHIMERA[39]. The density external to the capsid not accounted for by the P3 capsomers was interpreted as molecules of CPZ and modelled by MD simulations (see below).

Crystallization of P3 in complex with CPZ, data collection, and structure determination and refinement

Purified P3 protein was buffer exchanged into 10 mM Tris-HCl pH 8.5, 300 mM NaCl and concentrated to 5 mg/mL using a 10 kDa cut off Amicon concentrator device. Plates were then set up using the P3 trimer alone using previously described conditions[23]. Soaks were performed with 1 mM, 15 mM and 25 mM final concentrations of CPZ for 30 minutes. Diffraction data were collected at room temperature at beamline I24, Diamond Light Source (UK) using a 50 x 50 µm beam size, at either 50 % (native), or 100 % (soaked) transmission, with 0.1^{o} oscillation between frames, 0.01-0.05 s exposure time as a 360o wedge. Data were indexed, integrated, scaled and merged using the xia2-dials pipeline (Table S2†). As determined previously, all crystals were indexed with a $P2_12_12_1$ space group. Intensities were converted to structure factor amplitudes using TRUNCATE[45], before model refinement using PHENIX[46]. A round of rigid body refinement with the P3 trimer (PDB ID 1hx6) as initial model was performed prior positional and B-factor refinements with torsion-NCS restraints; the latter were relaxed in the last three refinement cycles (Table S2†).

Virus infectivity and thermal stability assays

Purified PRD1 particles (0.5 mg/ml) were treated with 30 or 60 mM CPZ in 20 mM potassium phosphate pH 7.2 or pH 6.0, 1 mM $MgCl_2$ for 15 min or 2 hrs at room temperature. Non CPZ-treated particles were used as a control. CPZ-treated PRD1 (30 mM CPZ) and control particles were either left in the buffer above or in the same buffer further supplemented with 150 mM or 300 mM NaCl (incubated for 30 min at 18 °C). Sedimentation of the particles was analysed by rate zonal centrifugation in 5-20% (w/v) sucrose gradient (20 mM potassium phosphate pH 6.0, 1 mM $MgCl_2$; Sorvall TH641 rotor, 24 000 rpm, 50 min, +15 °C). Specific infectivities of the light-scattering bands were calculated based on plaque assay data and absorbance measurements (NanoDrop). Protein composition was analysed by protein gel electrophoresis (16% w/v acrylamide) and Coomassie blue staining[47].

Solutions of PRD1 with or without 60 mM CPZ (CPZ solution prepared as above) at either pH 6.0 or pH 7.2 as well as CPZ alone at these two probe pHs were prepared and incubated at room temperature for 15 minutes before being put on ice. No discolouration, nor evidence of protein precipitation was observed for any of the samples.

Samples were then aliquoted into a 96-well PCR plate containing SYBR green DNA dye (Thermo Fisher Scientific) in triplicate, sealed and gently centrifuged for 3 min at 4 °C. The plate was then heated in a Mx3005p qPCR machine (Agilent Technologies, USA) from 25 to 97 °C in 1 °C min^{-1} increments for 30 s, with temperature cycles following an expanding saw-tooth profile, where fluorescence changes were monitored with exci/em 492/517 nm at the 25 °C 'troughs' of this saw-tooth profile[48].

Quantum mechanical calculations and molecular dynamic simulations of CPZ

For quantum mechanical calculations, full geometry optimizations of CPZ monomers, dimers and trimers excluding the *N,N*-dimethylpropanamine moiety tail were carried out with Gaussian 16 using the M06-2X hybrid functional and a 6-31+G(d,p) basis set with ultrafine integration grids[49]. Bulk solvent effects in water were considered implicitly through the IEF-PCM polarizable continuum solvent model[50]. The possibility of different conformations was considered for all structures. All stationary points were characterized by a frequency analysis performed at the same level used in the geometry optimizations from which thermal corrections were obtained at 298.15 K. The quasi-harmonic approximation reported by Ribeiro et al[51]. was used to replace the harmonic oscillator approximation for the calculation of the vibrational contribution to enthalpy and entropy. All computed structures can be obtained from authors upon request.

The QM-optimized geometries for CPZ monomers were used as starting coordinates for the Molecular Dynamics (MD) simulations of CPZ loading at the MCP P3 surface. Protein models were generated extracting 618 and 658 residues capsid fragments from the

 This journal is © The Royal Society of Chemistry 20xx

Please do not adjust margins

Please do not adjust margins

regions underlying the sphere-like and elongated density, respectively. For the protein model underlying the sphere-like density, only the V1 jellyroll of P3 was conserved (residues S35 to L240), for a total of three V1 jellyrolls belonging to different chains. For the model underlying the elongated density, a four- chains model was constructed containing two non-equivalent fragments of V1 and V2 jellyrolls corresponding to residues Y36 to Q239 and L257 to T381, respectively. Initial geometries for the CPZ-loaded capsid protein were generated by immersing protein models in a rectangular box of 3,200 CPZ molecules using the Packmol package[52]. Then, excess CPZ molecules were trimmed leaving an ellipsoidal (431 molecules) and a spherical (266 molecules) distribution to represent cigar-like and sphere-like densities, respectively. CPZ has a pKa of 8.6, meaning that its protonated (charged) and deprotonated (neutral) forms will coexist at pH 7.2[21]. As an approximation, the protonation state of CPZ was randomized to obtain an approximate 1:1 ratio of the two forms. MD simulations were carried out with the AMBER 20 package. The ff14SB and the general Amber force fields (GAFF2) were used to describe the protein and CPZ, respectively[53-55]. CPZ parameters were generated with the antechamber module of AMBER, using the GAFF2 force field and with partial charges set to fit the electrostatic potential generated with HF/6-31G(d) using the RESP method[56]. Charges were calculated according to the Merz-Singh-Kollman scheme using Gaussian 16[46]. Protein complexes were immersed in a water box with a 10 Å buffer of TIP3P water molecules and neutralized by adding explicit K^+ or Cl^- counterions[57]. A two-stage geometry optimization approach was performed. The first stage minimizes only the positions of solvent molecules and ions, and the second stage is an unrestrained minimization of all the atoms in the simulation cell. The systems were then heated by increasing the temperature from 0 to 300 K under a constant pressure of 1 atm and periodic boundary conditions. Harmonic restraints of 10 kcal/mol were applied to the solute, and the Andersen temperature coupling scheme was used to control and equalize the temperature[58,59]. The time step was kept at 1 fs during heating stages, allowing potential inhomogeneities to self-adjust. Water molecules were treated with the SHAKE algorithm such that the angle between the hydrogen atoms is kept fixed through the simulations[60]. Long-range electrostatic effects were modelled using the particle mesh Ewald method[61]. An 8 Å cut-off was applied to Lennard-Jones interactions. Each system was equilibrated for 2 ns with a 2 fs time step at a constant volume and temperature of 300 K. Production (400 ns) was run in the NVT ensemble imposing harmonic constraints on the alpha carbons of the protein models with the exception of the flexible loops in proximity to the CPZ aggregates (residues 47-54, 78-84, 217-223, 266-274, 292-303, 364-372 for the cigar-like density and 47-54, 78-84, 205-215, 217-223 for the sphere-like density). Squared atomic positional fluctuations were computed with the *cpptraj* module of AMBER.

The theoretical densities of CPZ molecules fitting the experimental sphere-like and cigar-like extra electronic densities were estimated from MD simulations. To this aim, a CPZ aggregate model consisting of 1000 CPZ molecules embedded in aperiodic box of water molecules was generated. The protonation state of each CPZ molecule was randomized to obtain a total 1:10 distribution of charged:neutral species. This system was subjected to MD simulation using the same protocol described above. Along the MD simulation, the CPZ aggregate assumes a spherical shape with charged CPZs lining the sphere's surface and neutral CPZs packed at the solvent-excluded volume. Once the system was equilibrated (*i.e.* when the cluster acquired a constant volume), different regions of the large CPZ aggregate was fitted into both extra-density isosurfaces (isovalues: sphere-like density = 0.0555 e- / $Å^{-3}$, cigar-like density = 0.0473 e- / $Å^{-3}$) using the Fit in map tool as implemented in Chimera software[41]. To obtain an average number of CPZ molecules from the heterogeneous molecular distribution of our aggregate model, five snapshots extracted from the MD simulation corresponding to the last 5 ns of the 400 ns simulation were selected. For each snapshot, five randomly selected regions of the aggregate were fitted into the extra-density isosurfaces, affording a total of 25 independent fittings for each extra-density shape. Averaging over these 25 values, a mean of 2.99 CPZ molecules for the sphere-like density (standard deviation = 0.25) and 8.39 for the cigar-like density (standard deviation = 0.37) were obtained. These values were calculated assuming that complete molecules reside within the extra electronic densities. Translating these values into densities required an estimation of the volume enclosed in the extra-density isosurfaces (~1260 $Å^3$ for sphere-like and ~3800 $Å^3$ for cigar-like densities, respectively), for which the Measure Volume and Area tool in Chimera was used. Considering the molecular mass of neutral CPZ (318.86 g mol^{-1}), theoretical densities ρ were derived for the two extra-density shapes. In both cases, we computed ρ for each number of CPZ molecules in each snapshot (25 independent fittings) using the following formula:

$$\rho = [(\text{n. CPZ molecules})/N_A] \times MW \times 1/V$$

where ρ is the density in g cm^{-3}, N_A is Avogadro's number, MW is the molecular weight in g mol^{-1} and V is the volume of the extra-density region in cm^3. The 25 density values were averaged and are presented with their standard deviation.

Please do not adjust margins

Please do not adjust margins

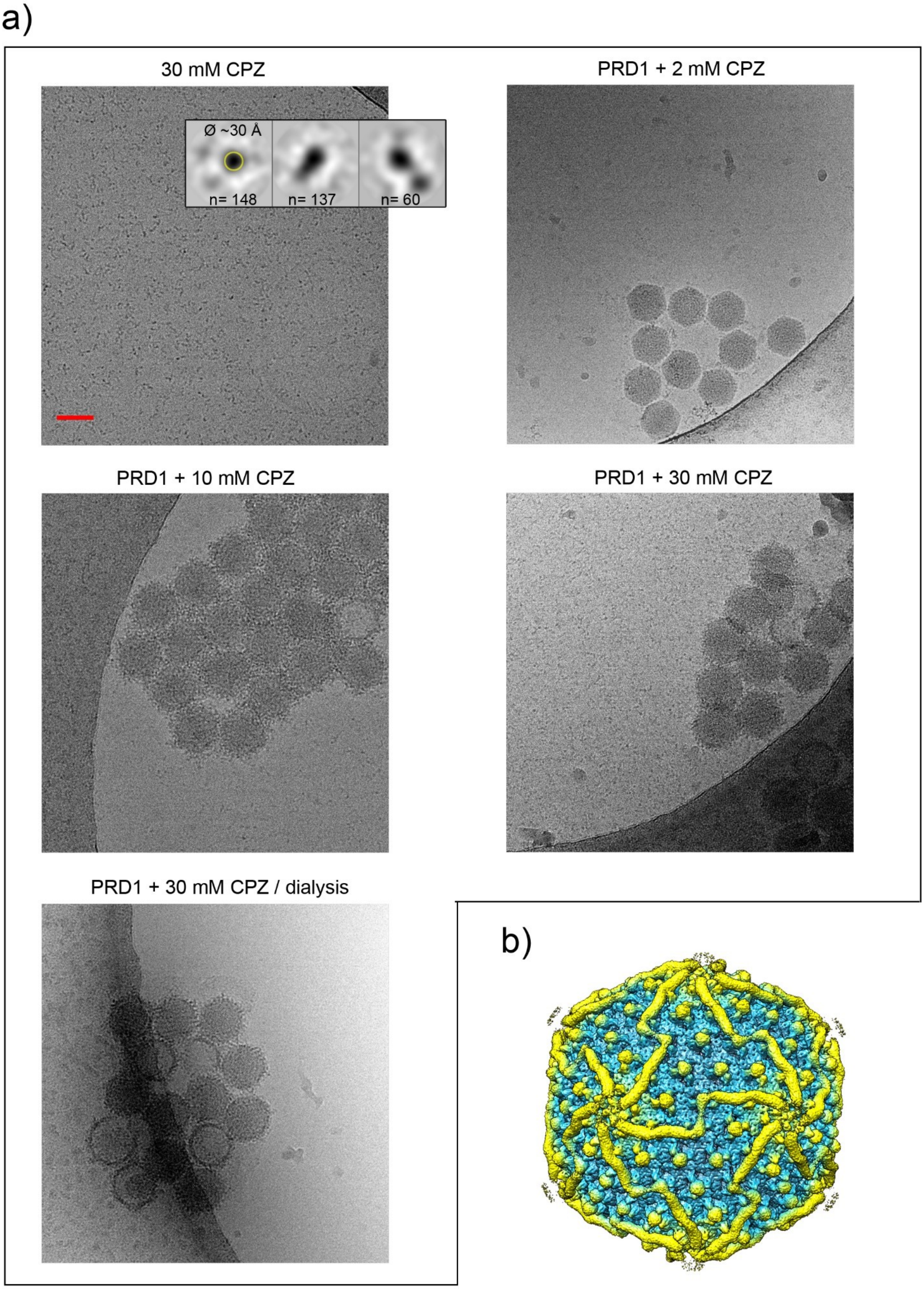

 This journal is © The Royal Society of Chemistry 20xx

Please do not adjust margins

Please do not adjust margins

Figure S1. 2D cryo-images and 3D electron density of CPZ-PRD1. (a) Comparison of 2D cryo-images of bacteriophage PRD1 in presence of different concentrations of CPZ (see also Fig. 1). The inset on top left panel shows three 2D class-averages of 345 'dot' particles autopicked in RELION software and then classified to roughly estimate the size. Scale bar, 50 nm; all micrographs are to scale. (b) Cryo-EM map as that in Fig. 2b but displayed at the lower threshold level of 0.01 in Chimera[39] to highlight the extent of the decoration.

Please do not adjust margins

Please do not adjust margins

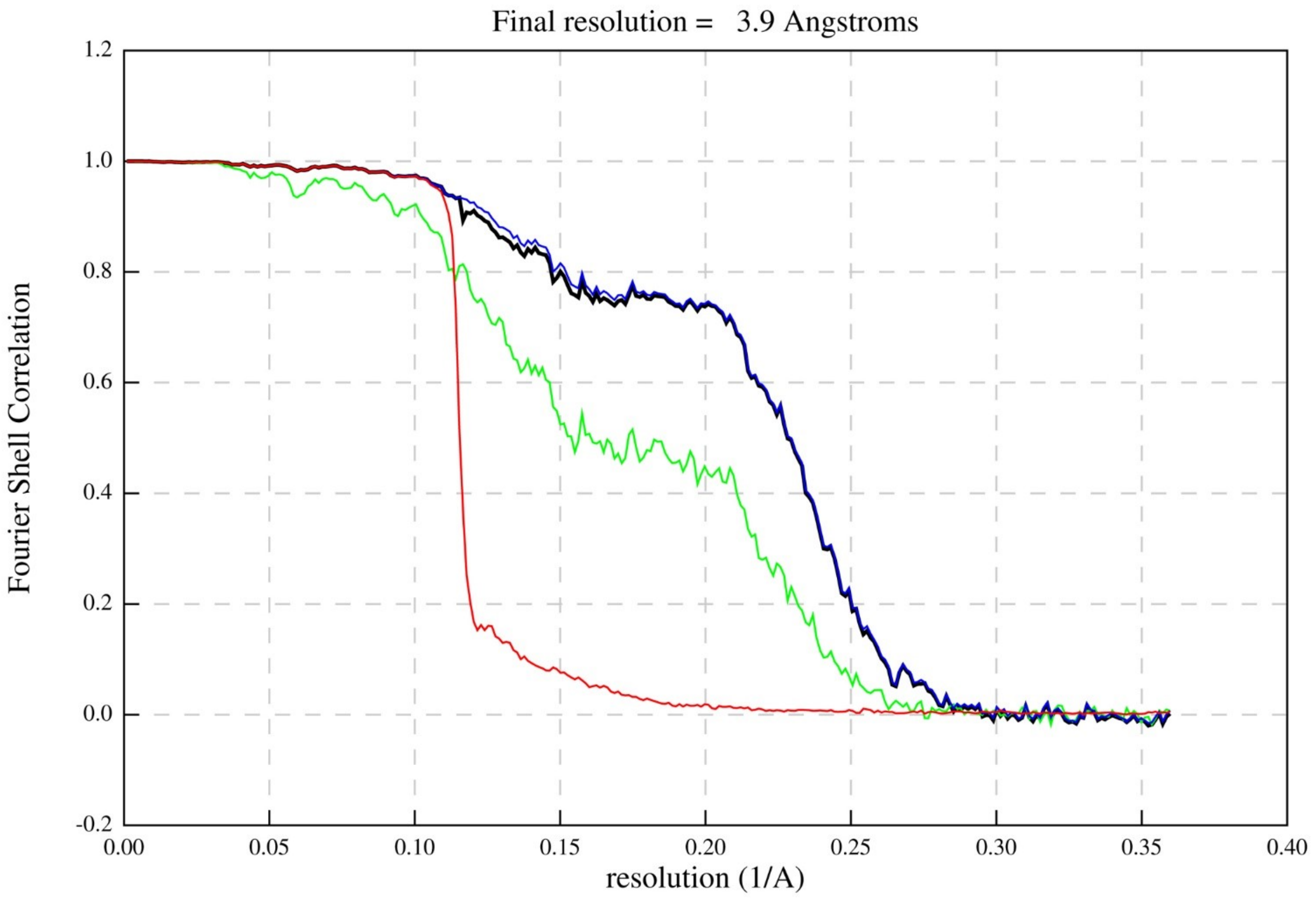

Figure S2. Fourier shell correlation (FSC) of the final CPZ-PRD1 3D reconstruction obtained using gold-standard refinement in RELION, marked with the 3.9 Å resolution corresponding to a FSC of 0.143. FSC is plotted for: masked maps (blue), corrected maps (black), phase randomized masked maps (red), and unmasked maps (green).

This journal is © The Royal Society of Chemistry 20xx

Please do not adjust margins

Please do not adjust margins

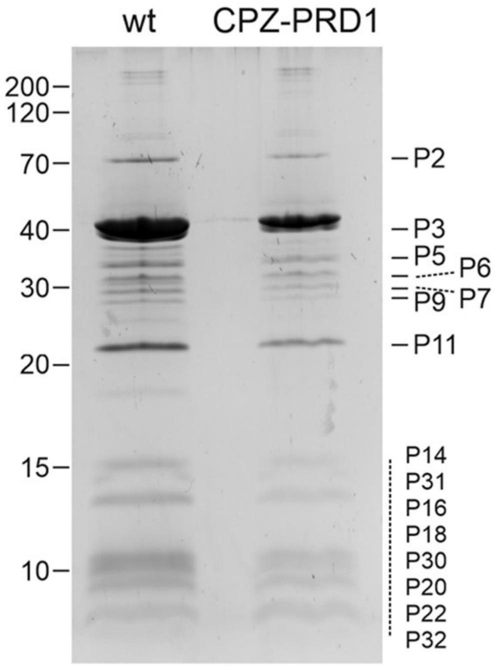

Figure S3. Analysis by SDS-PAGE of samples from rate zonal centrifugation (see Fig. 3a) showing that the protein composition of the CPZ-PRD1 is the same as the wild-type PRD1 used as control (untreated). Numbers on the left refer to the molecular weight of the marker while the letter on the right side indicate the viral proteins composing the virion.

Please do not adjust margins

Please do not adjust margins

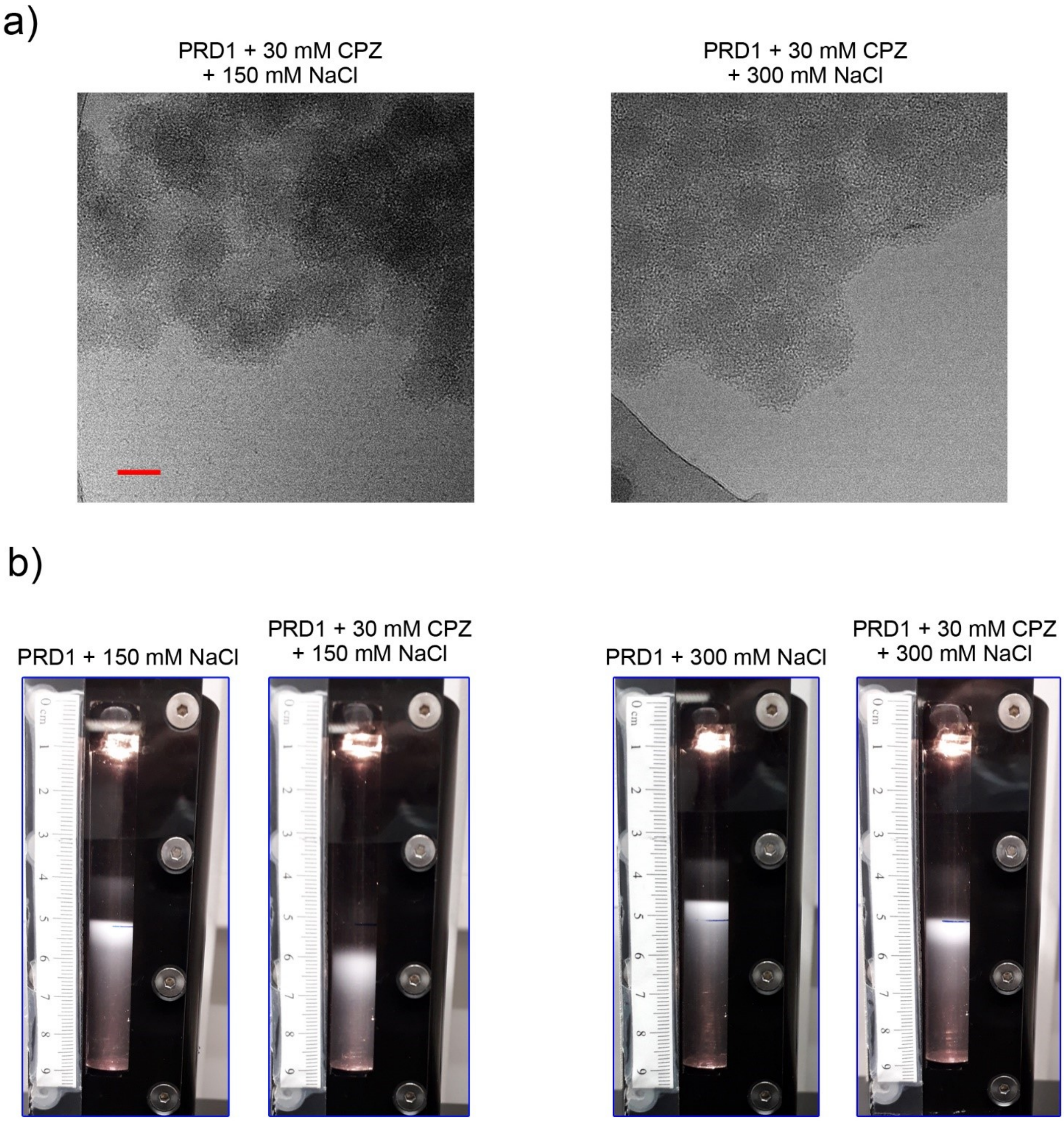

Figure S4. Effect of ionic strength on PRD1-CPZ particles. (a) Comparison of 2D cryo-images of bacteriophage PRD1 in presence of 30 mM CPZ and 150 and 300 mM NaCl, respectively (NaCl was added after preparing PRD1+CPZ solution). Scale bar, 50 nm; (b) Light-scattering zones after NaCl addition to PRD1 sample with and without CPZ and after rate zonal centrifugation in a sucrose gradient (PRD1 is known to be stable at 300 mM[62]).

 This journal is © The Royal Society of Chemistry 20xx

Please do not adjust margins

Please do not adjust margins

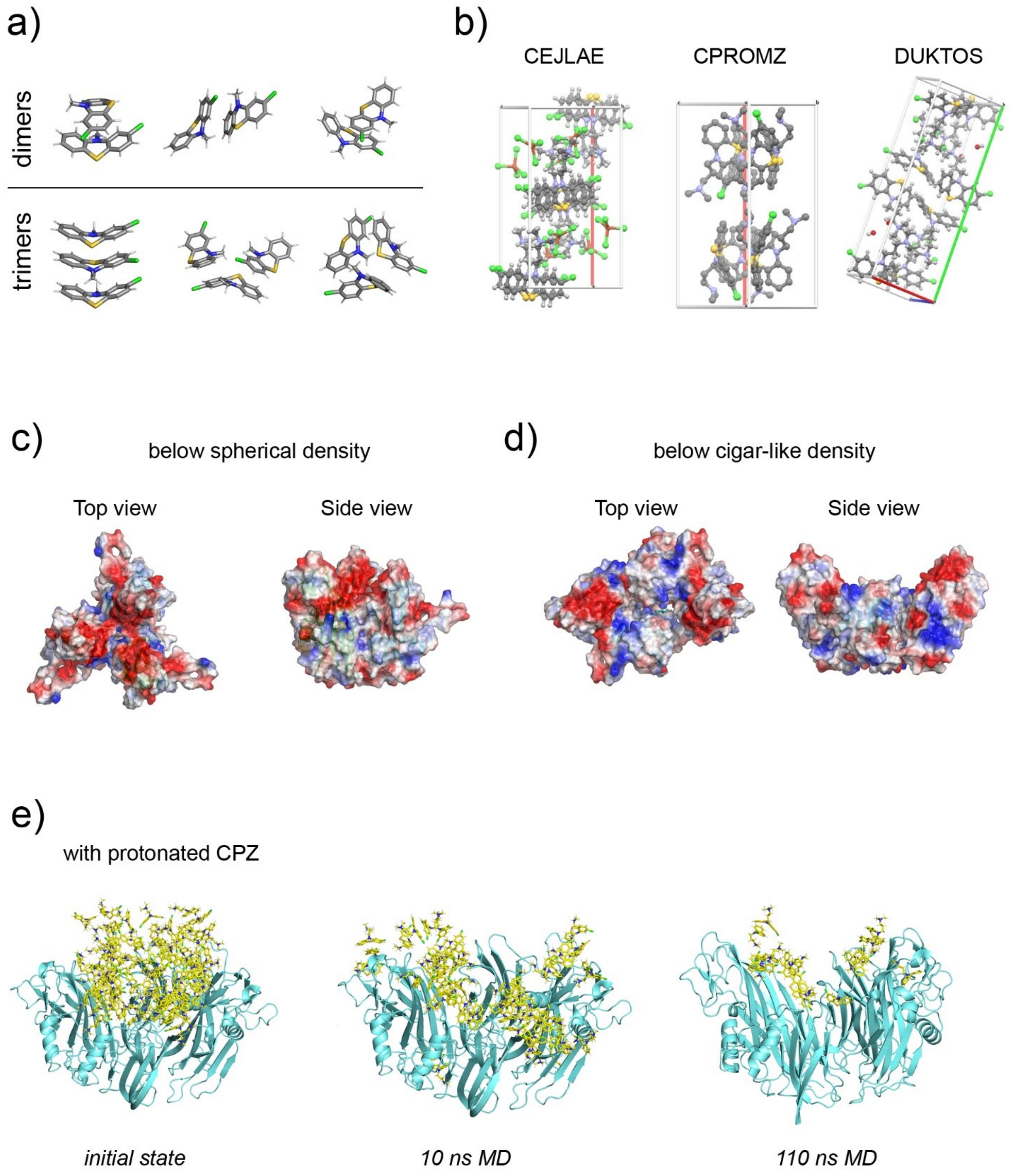

Figure S5. (a) Conformational space of the CPZ dimer and trimer explored by quantum mechanics but excluding the *N,N*-dimethylpropanamine moiety tail (see Experimental†). (b) Packing modes of CPZ experimentally determined by others by X-ray crystallography; the labels refer to their ID codes in the database, see Table S3†. (c) Representation of the isopotential surface of the three contiguous P3 V1 jellyrolls in close contact with the spherical extra electron density (red, negative; blue, positive). (d) Isopotential surface as (c) of the neighbouring P3 V1 and V2 jellyrolls contacting the cigar-like electron density. (e) Example of reorganization of CPZ molecules when considered 100% protonated at increasing simulation times (see Experimental†).

Please do not adjust margins

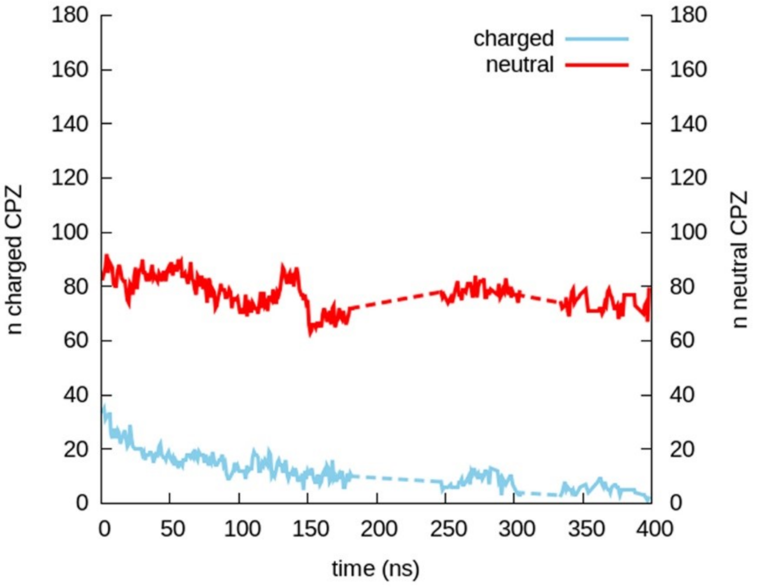

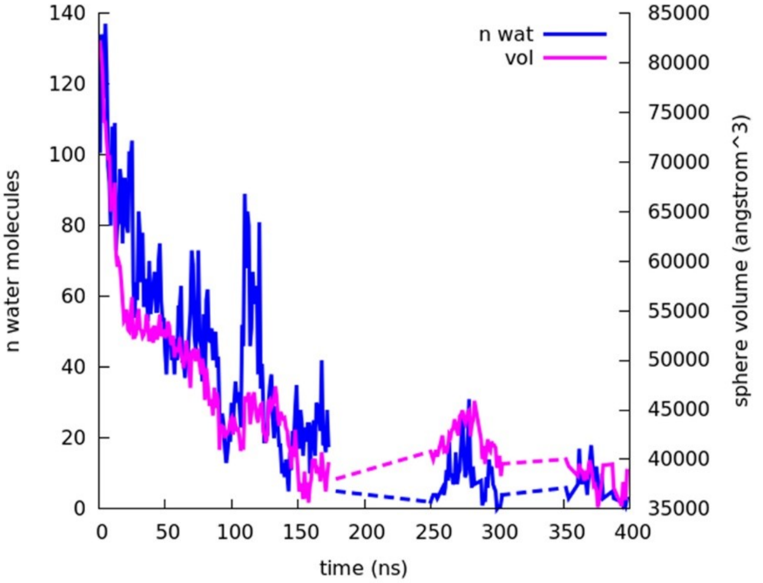

Figure S6. Sphere-like extra density modelled by MD simulations. Top, evolution of the number of charged (in blue) and neutral (in red) CPZ molecules at the core of the CPZ aggregate along the MD simulation. The number of charged CPZ molecules decreases sharply, indicating that already at 180 ns the aggregate core is mainly composed of neutral CPZ molecules. Bottom, evolution of the number of water molecules and size of the CPZ aggregate along the MD simulation. The aggregate shrinks as water molecules are excluded from its increasingly hydrophobic core. Missing points in the 170-250 and 300-350 ns ranges correspond to asymmetric CPZ molecules distributions that could not be fitted to a spherical surface.

 This journal is © The Royal Society of Chemistry 20xx

Please do not adjust margins

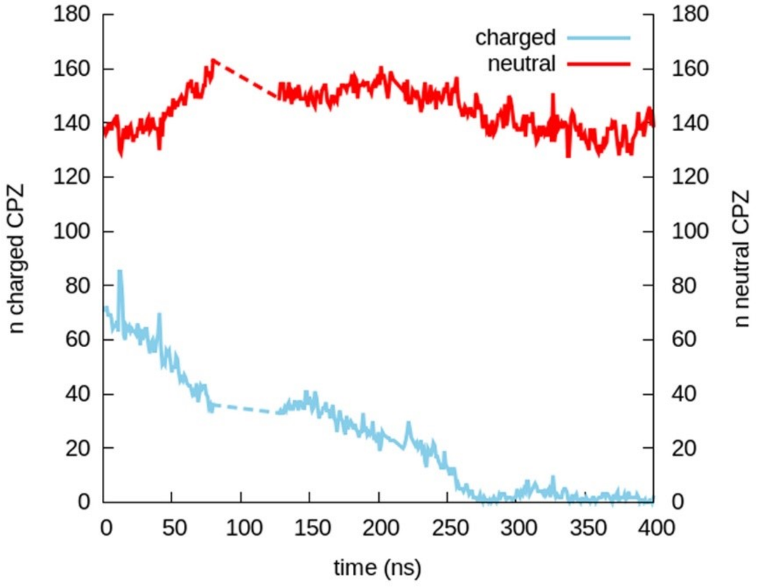

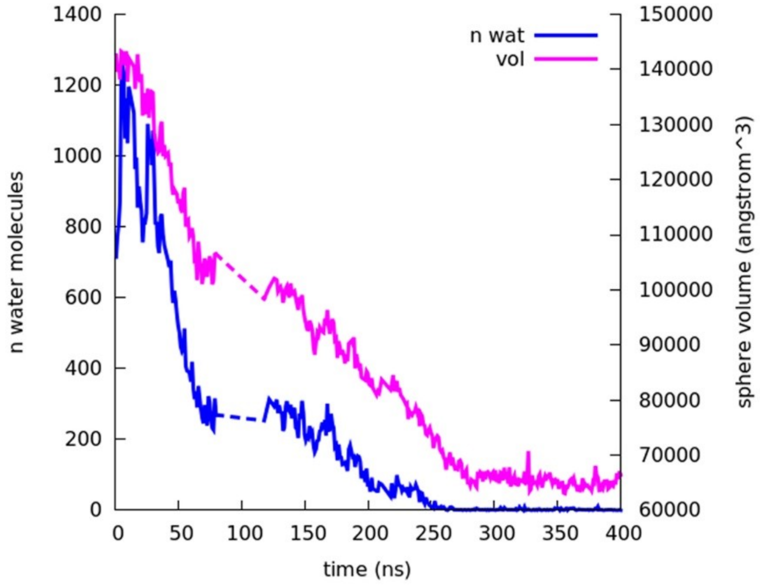

Figure S7. Cigar-like extra density modelled by MD simulations. Top, evolution of the number of charged and neutral CPZ molecules at the core of the CPZ aggregate along the MD simulation. The number of charged CPZ molecules decreases sharply, indicating that already at 180 ns the aggregate core is mainly composed of neutral CPZ molecules. Bottom, evolution of the number of water molecules and size of the CPZ aggregate along the MD simulation. The aggregate shrinks as water molecules are excluded from its increasingly hydrophobic core. Missing points in the 80-130 ns range correspond to asymmetric CPZ molecules distributions that could not be fitted to an elongated surface.

This journal is © The Royal Society of Chemistry 20xx

Please do not adjust margins

Please do not adjust margins

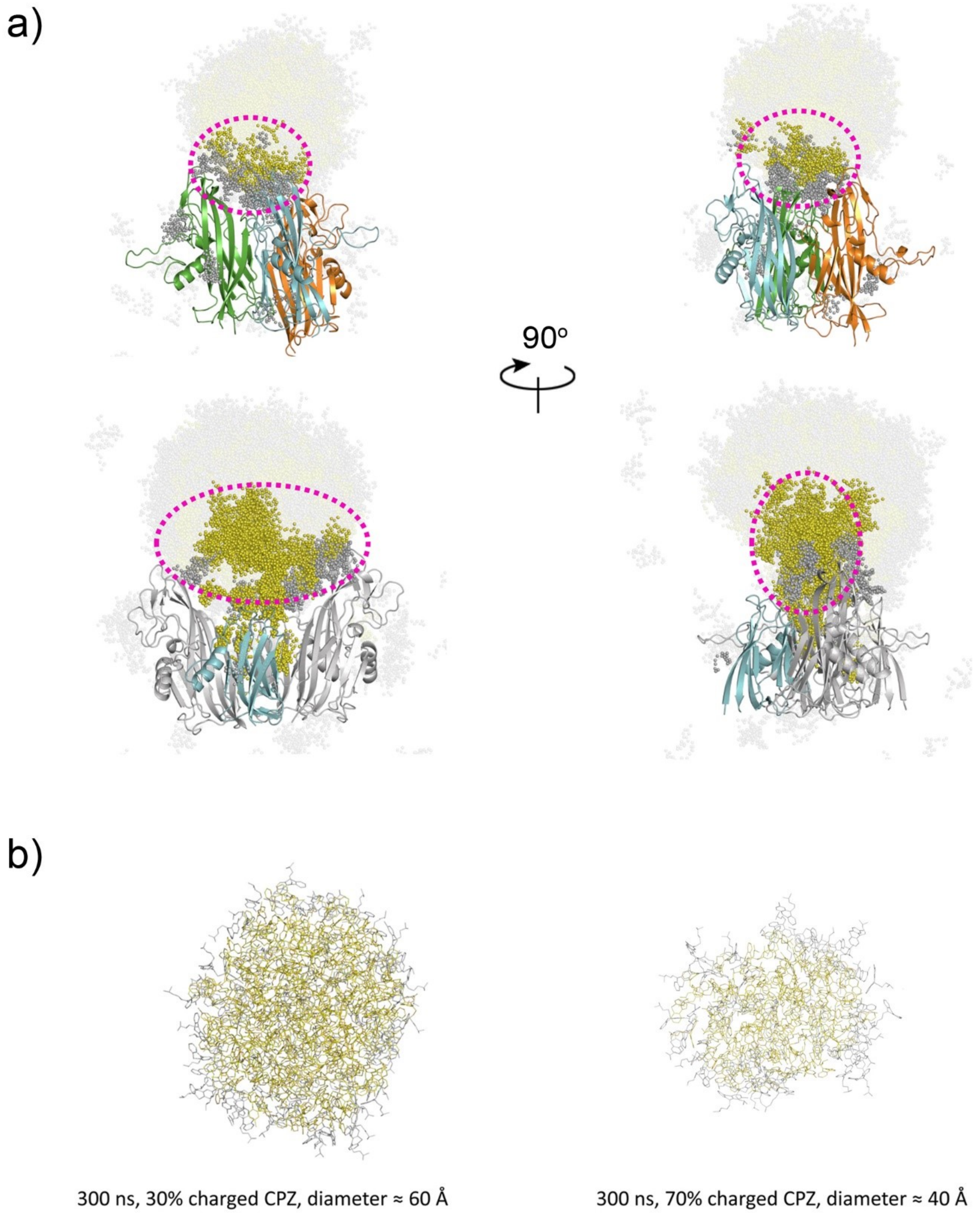

Figure S8. CPZ dynamics and size's dependence from percentage of charged molecules. (a) CPZ molecules with low mobility shown in yellow and grey spheres relative to the local molecular architecture (top, in the context of the adjacent three V1 jellyrolls; below, in the context of the V1 y V2 jellyrolls). (b) Micelle size's dependence from percentage of charged CPZ molecules.

 This journal is © The Royal Society of Chemistry 20xx

Please do not adjust margins

Please do not adjust margins

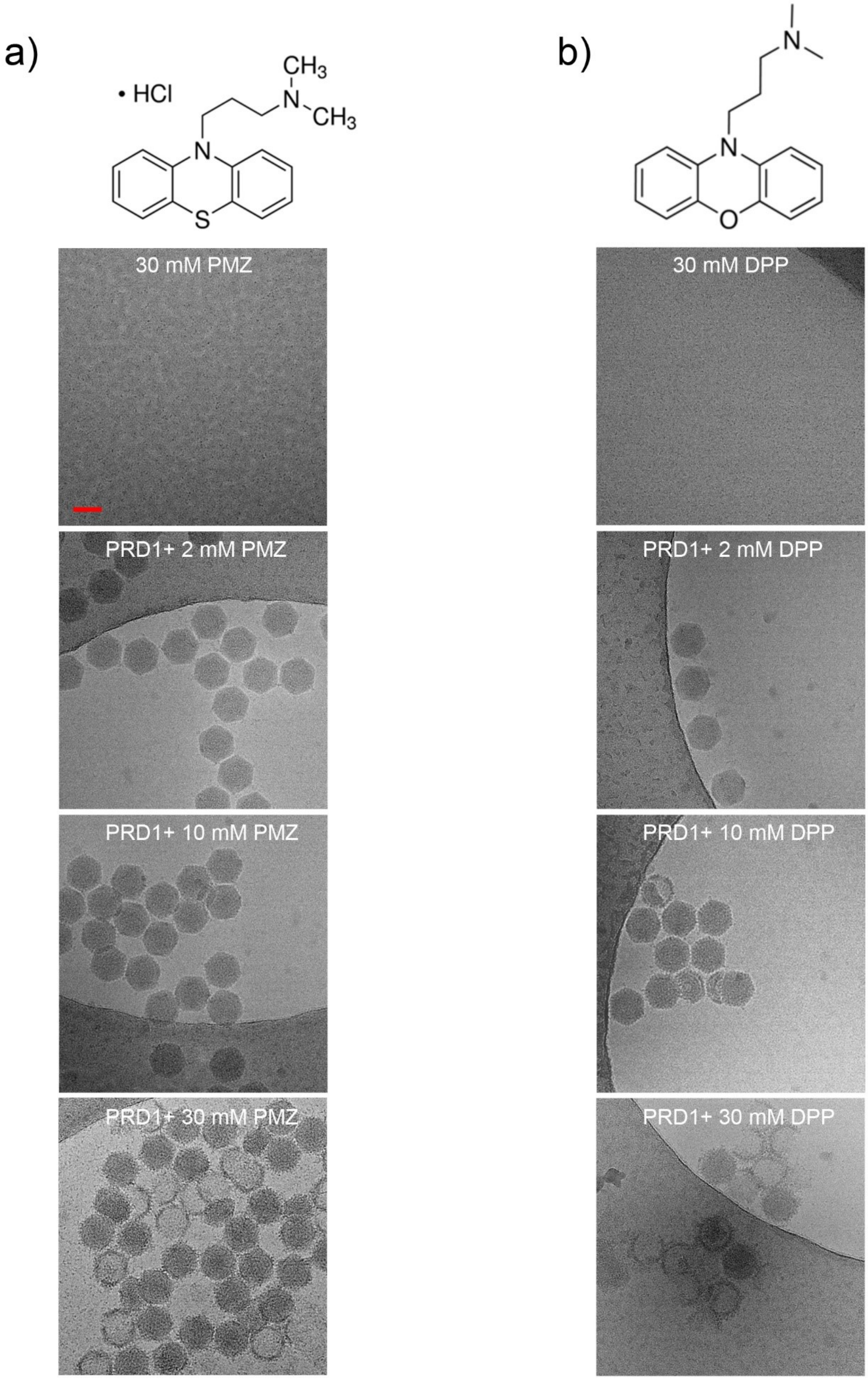

Figure S9. (a) Promazine hydrochloride (PMZ) structure (top) with below 2D cryo-images of PMZ alone and mixed with PRD1 at increasing drug concentrations. (b) Top, *N,N*-dimethyl-3-(10H-phenoxazin-10-yl)-1-propanamine (DPP) structure with below 2D cryo-images of DPP alone and mixed with PRD1 at increasing drug concentrations. All panels are to scale; scale bar, 50 nm.

Please do not adjust margins

Please do not adjust margins

Table S1. Cryo-EM data collection, refinement, and validation statistics for CPZ-PRD1

**Data collection**	
Nominal magnification	59,000
Voltage (kV)	300
Electron exposure (e^{-}/Å^2)	36
Defocus range (µm)	0.6-3.9
Sampling interval (Å/pixel)	1.39
Frames	27
Nº micrographs	3,218
**Cryo-EM processing**	
Contributing particles	3,414
Box Size (pixel)	768
Pixel size (Å)	1.4
Symmetry	I1
Map resolution (Å)<br>FSC threshold	3.9<br>0.143
Map sharpening *B* factor (Å^2)	-115
EMDB code	13109

 This journal is © The Royal Society of Chemistry 20xx

Please do not adjust margins

Please do not adjust margins

Table S2. X-ray data collection, refinement, and validation statistics of CPZ-P3 crystal structure

	**25 mM CPZ soaked into P3 crystal**
**Data Collection**	
Space Group	$P2_12_12_1$
Cell dimensions	
a, *b*, *c* (Å)	117.16 123.24 126.89
α, β, γ (°)	90, 90, 90
Wavelength (Å)	0.9686
Resolution (Å)	54.54 - 2.02<br>(2.09 - 2.02)*
R_{merge}	0.1148 (1.671)*
$CC_{1/2}$	0.999 (0.595)*
Mean I/σ(I)	14.03 (1.60)*
Completeness (%)	99.97 (100.00)*
Redundancy	13.1 (13.3)*
**Refinement**	
Resolution (Å)	39.83 - 2.23<br>(2.31 - 2.23)*
N° unique reflections	88837 (8694)*
R_{work}/ R_{free}	0.175 (0.267)*/ 0.215 (0.306)*
N° atoms	9308
Protein	8743
Ligand/ion	348
Water	414
B-factors	
Protein	48.81
Ligand/ion	74.48
Water	48.85
R.m.s. deviations	
Bond lengths (Å)	0.003
Bond angles (°)	0.572
Ramachandran plot	
Favoured (%)	97.30
Allowed (%)	2.70
Outliers (%)	0.00
PDB ID	7OOK

*Values in parenthesis refer to the highest resolution bin

This journal is © The Royal Society of Chemistry 20xx

Please do not adjust margins

Please do not adjust margins

Table S3. Deposited coordinates of CPZ in the Cambridge Crystallographic Data Centre (CCDC)

Deposition number	Database ID	Space group (#)	a	b	c	α ,β,γ	Stacking	Additive
1122637	CEJLAE	P n m a (62)	23.745	13.897	12.580	90,90,90	pi-pi	Cu(II)Cl_4
1131411	CPROMZ	P b c a (61)	23.50	15.20	9.23	90,90,90	1 up one down	
1131412	CPROMZ01	P b c a (61)	15.20	23.53	9.27	90,90,90		
1146386	DUKTOS	P 21/c (14)	11.861	31.671	9.599	90,90,90	Aminopropyl	with Cl^- and HOH
1318566	ZZZFHC	P 21/c (14)	11.99	32.33	9.89	90,90,90		with Cl^-

 This journal is © The Royal Society of Chemistry 20xx

Please do not adjust margins

Please do not adjust margins

Table S4. Sphere-like extra density

Number of neutral and protonated CPZ molecules in the first solvation shell of selected residues of P3 after 400 ns of molecular dynamics. We observe more contacts with charged than with neutral CPZ molecules. This matches the observation that protonated CPZ molecules line the exterior of the aggregate and form attractive electrostatic interactions with negatively charged residues such as D and E.

Residue	n. CPZ < 6 Å neutral	n. CPZ < 6 Å protonated
N79	1	3
T82	3	4
E83	0	3
D222	1	5
E224	1	5
E226	0	4
E227	0	2

Table S5. Cigar-like extra density.

Number of neutral and protonated CPZ molecules in the first solvation shell of selected residues of P3 after 400 ns of molecular dynamics. We observe more contacts with charged than with neutral CPZ molecules. This matches the observation that protonated CPZ molecules line the exterior of the aggregate and form attractive electrostatic interactions with negatively charged residues such as D and E.

Residue	n. CPZ < 6 Å neutral	n. CPZ < 6 Å protonated
D48	0	3
T77	3	3
N79	3	2
D149	1	0
T159	2	3
E161	1	2
D222	0	2
E224	0	3
E226	2	3
E227	1	1

This journal is © The Royal Society of Chemistry 20xx

Please do not adjust margins

Please do not adjust margins

Movie S1. CPZ modelling in the sphere-like extra density

Molecular dynamics simulation (400 ns) of 266 CPZ molecules (50:50 mixture of charged and neutral molecules) in contact with the protein model representing the capsid region underlying the spherical extra-density. CPZ molecules initially solvate three V1 jellyrolls. Along the simulation, most of the charged CPZ molecules diffuse into solution. The remaining charged CPZ molecules line a spherical aggregate of neutral CPZ sitting on the three V1 jellyrolls. Charged CPZ molecules are represented in grey, neutral CPZ molecules in yellow.

Movie S2. CPZ modelling in the elongated extra density

Molecular dynamics simulation (400 ns) of 431 CPZ molecules (50:50 mixture of charged and neutral molecules) in contact with the protein model representing the capsid region underlying the elongated extra-density. CPZ molecules initially solvate two V1 jellyrolls. Along the simulation, most of the charged CPZ molecules diffuse into solution. The remaining charged CPZ molecules surround a spherical aggregate of neutral CPZ sitting between the two V1 jellyrolls. Charged CPZ molecules are represented in grey, neutral CPZ molecules in yellow.

 This journal is © The Royal Society of Chemistry 20xx

Please do not adjust margins

Please do not adjust margins

Notes and references

40. J. K. Bamford, D. H. Bamford, Large-scale purification of membrane-containing bacteriophage PRD1 and its subviral particles. *Virology*, 1991, **181**, 348.
41. J. Caldentey, C. Luo, D. H. Bamford, Dissociation of the lipid-containing bacteriophage PRD1: effects of heat, pH, and sodium dodecyl sulfate. *Virology*, 1993, **194**, 557.
42. S. Q. Zheng et al., MotionCor2: anisotropic correction of beam-induced motion for improved cryo-electron microscopy. *Nat Methods*, 2017, **14**, 331.
43. A. Rohou, N. Grigorieff, CTFFIND4: Fast and accurate defocus estimation from electron micrographs. *J Struct Biol*, 2015, **192**, 216.
44. J. Zivanov et al., New tools for automated high-resolution cryo-EM structure determination in RELION-3. *eLife*, 2018, **7**.
45. N. Collaborative Computational Project, The CCP4 suite: programs for protein crystallography. *Acta Crystallogr D Biol Crystallogr*, 1994, **50**, 760.
46. P. V. Afonine et al., Real-space refinement in PHENIX for cryo-EM and crystallography. Acta Crystallogr D Struct Biol, 2018, **74**, 531.
47. V. M. Olkkonen, D. H. Bamford, Quantitation of the adsorption and penetration stages of bacteriophage phi 6 infection. *Virology*, 1989, **171**, 229.
48. T. S. Walter et al., A plate-based high-throughput assay for virus stability and vaccine formulation. *J Virol Methods*, 2012, **185**, 166.
49. M. J. Frisch, G. W. Trucks, H. B. Schlegel, G. E. Scuseria, M. A. Robb, J. R. Cheeseman, G. Scalmani, V. Barone, G. A. Petersson, H. Nakatsuji, X. Li, M. Caricato, A. Marenich, J. Bloino, B. G. Janesko, R. Gomperts, B. Mennucci, H. P. Hratchian, J. V. Ortiz, A. F. Izmaylov, J. L. Sonnenberg, D. Williams-Young, F. Ding, F. Lipparini, F. Egidi, J. Goings, B. Peng, A. Petrone, T. Henderson, D. Ranasinghe, V. G. Zakrzewski, J. Gao, N. Rega, G. Zheng, W. Liang, M. Hada, M. Ehara, K. Toyota, R. Fukuda, J. Hasegawa, M. Ishida, T. Nakajima, Y. Honda, O. Kitao, H. Nakai, T. Vreven, K. Throssell, J. A. Montgomery, Jr., J. E. Peralta, F. Ogliaro, M. Bearpark, J. J. Heyd, E. Brothers, K. N. Kudin, V. N. Staroverov, T. Keith, R. Kobayashi, J. Normand, K. Raghavachari, A. Rendell, J. C. Burant, S. S. Iyengar, J. Tomasi, M. Cossi, J. M. Millam, M. Klene, C. Adamo, R. Cammi, J. W. Ochterski, R. L. Martin, K. Morokuma, O. Farkas, J. B. Foresman, and D. J. Fox, Gaussian, Inc., Wallingford CT, 2016.
50. G. Scalmani, M. J. Frisch, Continuous surface charge polarizable continuum models of solvation. I. General formalism. *J Chem Phys*, 2010, **132**, 114110.
51. R. F. Ribeiro, A. V. Marenich, C. J. Cramer, D. G. Truhlar, Use of solution-phase vibrational frequencies in continuum models for the free energy of solvation. *J Phys Chem B*, 2011, **115**, 14556.
52. L. Martinez, R. Andrade, E. G. Birgin, J. M. Martinez, PACKMOL: a package for building initial configurations for molecular dynamics simulations. *J Comput Chem*, 2009, **30**, 2157.
53. K. B. D.A. Case, I.Y. Ben-Shalom, S.R. Brozell, D.S. Cerutti, T.E. Cheatham, III, V.W.D. Cruzeiro, T.A. Darden, R.E. Duke, G. Giambasu, M.K. Gilson, H. Gohlke, A.W. Goetz, R. Harris, S. Izadi, S.A. Izmailov, K. Kasavajhala, A. Kovalenko, R. Krasny, T. Kurtzman, T.S. Lee, S. LeGrand, P. Li, C. Lin, J. Liu, T. Luchko, R. Luo, V. Man, K.M. Merz, Y. Miao, O. Mikhailovskii, G. Monard, H. Nguyen, A. Onufriev, F.Pan, S. Pantano, R. Qi, D.R. Roe, A. Roitberg, C. Sagui, S. Schott-Verdugo, J. Shen, C. Simmerling, N.R.Skrynnikov, J. Smith, J. Swails, R.C. Walker, J. Wang, L. Wilson, R.M. Wolf, X. Wu, Y. Xiong, Y. Xue, D.M. York and P.A. Kollman. (University of California, San Francisco, 2020).
54. J. A. Maier et al., ff14SB: Improving the Accuracy of Protein Side Chain and Backbone Parameters from ff99SB. *J Chem Theory Comput*, 2015, **11**, 3696.
55. J. Wang, R. M. Wolf, J. W. Caldwell, P. A. Kollman, D. A. Case, Development and testing of a general amber force field. *J Comput Chem*, 2004, **25**, 1157.
56. C. I. Bayly, P. Cieplak, W. Cornell, P.A. Kollman, A well-behaved electrostatic potential based method using charge restraints for deriving atomic charges: the RESP model. *J. Phys. Chem.*, 1993, **97**, 10269.
57. W. L. Jorgensen, J. Chandrasekhar, J.D. Madura, R.W. Impey, M.L. Klein, Comparison of simple potential functions for simulating liquid water. *J. Chem. Phys.*, 1983, **79**, 926.
58. H. C. Andersen, Molecular dynamics simulations at constant pressure and/or temperature. *J. Chem. Phys.*, 1980, **72**, 2384.
59. T. A. Andrea, W.C. Swope, H.C. Andersen, The role of long ranged forces in determining the structure and properties of liquid water. *J. Chem. Phys.*, 1983, **79**, 4576.
60. S. Miyamoto, P.A. Kollman, Settle: An analytical version of the SHAKE and RATTLE algorithm for rigid water models. *J. Comput. Chem.*, 1992, **13**, 952.
61. T. Y. Darden, D. York, L. Pedersen, Particle mesh Ewald: An N·log(N) method for Ewald sums in large systems. *J. Chem. Phys.*, 1993, **98**, 10089.
62. H. M. Oksanen, A. Domanska, D. H. Bamford, Monolithic ion exchange chromatographic methods for virus purification. *Virology*. 2012, **434**, 271.

Please do not adjust margins
